# The early rise and spread of evolutionary game theory: perspectives based on recollections of early workers

**DOI:** 10.1098/rstb.2021.0493

**Published:** 2023-05-08

**Authors:** Jean-Baptiste Grodwohl, Geoff A. Parker

**Affiliations:** ^1^ Department of History and Philosophy of Science, Laboratoire SPHERE, UMR7219, University of Paris Cité, Paris 75 013, France; ^2^ Department of Evolution, Ecology and Behaviour, University of Liverpool, Liverpool L69 7ZB, UK

**Keywords:** evolutionarily stable strategy, John Maynard Smith, history of evolutionary game theory

## Abstract

Though the first attempts to introduce game theory into evolutionary biology failed, new formalism by Maynard Smith and Price in 1973 had almost instant success. We use information supplied by early workers to analyse how and why evolutionary game theory (EGT) spread so rapidly in its earliest years. EGT was a major tool for the rapidly expanding discipline of behavioural ecology in the 1970s; each catalysed the other. The first models were applied to animal contests, and early workers sought to improve their biological reality to compare predictions with observations. Furthermore, it was quickly realized that EGT provided a general evolutionary modelling method; not only was it swiftly applied to diverse phenotypic adaptations in evolutionary biology, it also attracted researchers from other disciplines such as mathematics and economics, for which game theory was first devised. Lastly, we pay attention to exchanges with population geneticists, considering tensions between the two modelling methods, as well as efforts to bring them closer.

This article is part of the theme issue ‘Half a century of evolutionary games: a synthesis of theory, application and future directions’.

## 1. Introduction

The success of evolutionary game theory (EGT) since Maynard Smith and Price published their classic 1973 *Nature* paper ‘The logic of animal conflict’ [1] is well known. In the span of just a few years, it became one of the main modelling methods in the study of phenotypic evolution. It has stimulated numerous naturalists who endeavoured to use it to interpret their data. Now a genuine theory, i.e. a field of mathematical research that has enriched the other branches of game theory, it is taught in many academic programmes in theoretical biology, evolutionary biology and animal behaviour.

When a method is almost instantly used, discussed and applied by several practitioners, its success is seen as unsurprising, obvious. The theory was so ‘right’, so interesting, that one comes to think that it *had* to spread. One only needs to celebrate the initiators for coining such a useful approach; the rest, as they say, is history. But there was nothing obvious in the rapid success of EGT. With hindsight, it is surprising that naturalists found in mathematical models and simplified computer simulations a stimulus for novel reinterpretations of their data, or for starting new work. And also remarkable that such diverse theoreticians—mathematicians, physicists, economists and biologists—decided to use these models, creating a field of theoretical research.

Here, we have interviewed a cast of contributors to ascertain how they became informed of the opportunities of EGT for their own work. Our intent is to pay attention both to networks of information, and to EGT's burgeoning effect on research programmes, theoretical and mathematical. In reconstructing these developments, we pay much attention to the effective influence of John Maynard Smith, who remained, for the span of a decade or more, the main contributor, the main popularizer, and, to use a term he employed in another context, the main marriage-broker among theoreticians and naturalists. But we also hope to go beyond the appreciation of the contributions of a single researcher, which have rightly been celebrated (e.g. [2–7]). EGT can be seen as a success because a community took shape that studied and used it: here, we put its emergence under closer focus. Since one of us (G.A.P.) was involved in these developments, we will draw, often extensively, on his recollections and subjective appreciation on developments and events; for this reason, G.A.P. is referred to throughout as ‘I’, ‘me’, etc. in this article.

## 2. Evolutionary game theory: first steps

The reception given to the theory of games developed by von Neumann and Morgenstern [8] in the mid-1940s provides a useful starting point. Albeit surprisingly, but abundantly documented by historians, game theory met with only lukewarm interest among its intended audience of economists (e.g. [9,10]). The first researchers to make extensive use of game theory were applied mathematicians working in the new institutes of Cold War Science, especially the RAND Corporation, founded in 1948 as an advisory committee in research and development for the U.S. Armed Forces. Thus, game theory's initial success did not lie in fuelling the interest of economists, but in its ability to offer tools to other communities of investigators. One of the most instructive works on this history is Paul Erickson's *The world the game theorists made* [11], which reconstructs the circulation of game theory in several scientific communities over the course of the Cold War. The Cold War provided the major context for both the motivation and the funding for research on game theory. Applied mathematicians found in it a set of convenient optimizing methods for their modelling decisions in situations of uncertainty. Then, from the mid-1950s onwards, directly stimulated by the immediate context of the Cold War, researchers in social and political sciences adopted these methods to study conflicts and their resolutions.

A fascinating chapter in Erickson's book [11] concerns the way evolutionary biologists adopted game theory: it was not an instant success. Erickson draws a sharp contrast between the theory of games as promoted by the first generation of population biologists in the United States in the 1960s, such as Lewontin in 1961 [12], and Slobodkin in 1964 [13], with the evolutionary theory of games fashioned by Hamilton [14] and Maynard Smith and Price [1] a few years later in the UK. Both groups used game theory, but the former used it mainly as an analogy, which proved to be less fruitful than they initially hoped. Thus understood, game theory invaded evolutionary biology in two different waves, stimulated by very different theoretical aims, and which met very different fates. The main difference between these approaches was the scale at which selection was assumed to act.

In the first wave, populations were pictured as having strategies against the environment. Lewontin's paradigmatic 1961 game theory paper pictured genetic polymorphism as a randomizing strategy played against a changing environment. This analogy was interesting, even striking, but it failed to generate any research programme. Further, the environment can hardly be envisaged as a strategic player. Even Lewontin became unenthusiastic that it could be a useful tool in evolution [11,15]. A more compelling example was given by R. A. Fisher in 1958 [16], who suggested that genetic polymorphism could represent a mixed strategy in an evolutionary game against predators. This suggestion was never adopted, and only served, in later accounts, as a forerunner to the evolutionary theory of games.

By contrast, as admirably recapitulated by Erickson [11], the second wave of evolutionary game theorists, Hamilton, Maynard Smith and Price, made it a modelling method tailored for phenotypic selection: they applied game theory in terms of the behaviour of *individuals*. There were several examples where researchers, especially at the start of the behavioural ecology era in the late 1960s and early 1970s were considering cases where individual fitness depended on both one's own action and the actions of others in the same population (e.g. see [17]). Some of us clearly felt the need for an approach studying selection pressures occurring simultaneously on the same individuals. For example, the requirement for such a formalism was stated explicitly in a letter to me by Robert Trivers (R. L. Trivers 1971, personal communication to G.A.P.; see [18]):

Someday, particularly for social traits, we will have to work out some more formal principles for applying natural selection than are commonly employed: you routinely think in terms of selection pressures operating simultaneously on several individuals at the same time, but this is not common, and it should be of value someday for someone to formulate in detail working rules by which one makes sophisticated functional arguments.

Maynard Smith and Price's [1] central breakthrough was to propose just that—a technique for analysis. They envisaged animals as players adopting strategies in an evolutionary game and sought an evolutionarily stable strategy (ESS), i.e. a strategy that, when played by the population, could not be invaded by any rare mutant strategy. While simple optimization was inadequate, their two ESS conditions permitted a form of competitive optimization suitable for analysis of inter-individual conflicts: thus EGT and behavioural ecology grew together rapidly and were synergistic, each both necessary for and simultaneously catalysing the spread of the other.

## 3. John Maynard Smith, George Price and the evolutionary theory of games

Maynard Smith's contribution has sometimes been downplayed, in suggestions that his role was limited to disseminating and popularizing a method invented by more creative minds, first among them W. D. Hamilton and G. R. Price, in the late 1960s. A highly distinguished, recently deceased ecologist once told me of his feeling that Maynard Smith's notable talent consisted of his sharp clarity in developing and making use of insights. It is not our intention here to challenge this appreciation by reviewing Maynard Smith's numerous creative contributions throughout his career. We limit ourselves to demonstrating how the growth of EGT as a modelling method in the 1970s was simply inseparable from Maynard Smith's inputs.

Maynard Smith was not only the co-author of the 1973 paper [1] that founded EGT. Over the decade that followed its publication, he remained the main force for its growth, through both his scientific works and his ability to attract and stimulate talents. Without Maynard Smith, similar modelling methods for studying frequency-dependent selection at the phenotypic level would almost certainly have been developed: game-like approaches in newly emerging behavioural ecology (e.g. [19,20]), sex ratio theory [14,21] and anisogamy evolution [22] show that researchers working on interacting phenotypes needed such a method. But Maynard Smith's contribution had been precisely this: he and Price had gone beyond tackling a single problem (the evolution of animal conflicts) to generate an analytical method that could be applied to any phenotypic situation involving frequency-dependent selection.

Much has been written on the collaboration between Maynard Smith and Price that generated ESS formalism [15,23–25]. Prompted by Hamilton's papers on the evolution of altruism, Price became interested in evolutionary theory and set out to construct a method for modelling the evolution of altruistic traits. In parallel, he investigated how strategies limiting damage in conflicts could evolve, resulting in a long manuscript, ‘Antlers, intraspecific combat and altruism’, submitted to *Nature*. Maynard Smith refereed ‘Antlers’, and wrote a favourable report suggesting cuts, and Price put his manuscript aside. Stimulated, Maynard Smith began taking an interest in the subject. In a sabbatical at the University of Chicago in autumn 1970, he mentioned to students his problems in convincing Price to publish his results (M. Slatkin 2011, personal communication to J.B.G.). Maynard Smith eventually published a few pages on the method in a popular book *On evolution* before the publication of their joint paper; the acknowledgements attributed the credit of the idea to ‘Dr. George Price, now working in the Galton Laboratory at University College London. Unfortunately, Dr Price is better at having ideas than at publishing them’ [26, pp. vii–viii].

The long delay between Price's original submission and the publication of the joint paper deserves comment. Price wanted to improve his manuscript, met some problems in computer simulations (his main problem was to find strategies resisting small perturbations) and gradually set the project aside. This reflected a wider pattern characterizing Price's brief career as a theoretical biologist. After writing a text, Price could quickly lose interest in it and turn to something else. A grant application he wrote in 1969 reflects the tremendous diversity of his research interests, from altruism to sexual selection (G. R. Price, ‘Proposal to the Science Research Council’, Supplementary Details of Intended Research: On group selection, human evolution’, GRPP 84116; see [23]). But this boundless curiosity, and the intensity he brought to any problem under his consideration, had as a reverse side an ability to become detached from a problem once he had worked on it. This was indeed the only reservation Hamilton made when writing a report on Price's application: ‘there seem[ed] just a possibility that he might lose interest in the work halfway through, not care to publish results, not heed biological advice as to what were reasonable models, or some such thing’ (Hamilton to P. H. Williams, Secretary of the Biological Sciences Committee, SRC, 2 May 1969, GRPP^1^ 84116). With hindsight, Hamilton's intuition was remarkably prescient.

A case in point is Price's dealings with his major methodological contribution, the equation that now bears his name. This equation [27] has been justly celebrated (e.g. [28]) and has proved to be a very powerful guide for framing problems in evolutionary theory (see [23]). However, tellingly, by 1973 Price was already disappointed with his own contribution. The Galton Laboratory was then producing reams of electrophoretic data on enzyme variation in humans. Now interested in the problems raised by enzyme variation, Price turned his interests to statistical tests of neutrality (e.g. [29]). His equation turned out to be of little help, and Price mentioned his disillusionment to Hamilton: the equation was less useful than he thought, since it did not distinguish between selection pressure and population properties (Price to Hamilton, 13 August 1973, WDHP, Z1 X 83). The subsequent history of the Price equation would deserve a separate paper; the equation was not an instant success. In his autobiography, Hamilton has explained how he managed to get Price's note published in *Nature* [30]; he himself used Price's method in his papers, in his lectures at UCL and, later, at the University of Michigan, but to limited immediate effect—few students seemed to appreciate it. By the late 1970s, Hamilton was wondering if finding usefulness in that approach reflected some mental twist peculiar to Price and himself (Hamilton to Jon Seger, 12 February 1981, WDHP, Z1 X 63; on Hamilton and Price's collaboration, see [31]). It was only in the early 1980s, when Seger, then a PhD student at Harvard, made use of it to model coefficients of relatedness in kin selection research, that the Price equation took life as a modelling tool [32]. So if the fate of EGT had rested solely upon Price's shoulders, would the ‘Antlers’ paper have been shortened to the point of being almost unusable (just as with his terse 1970 note in *Nature* introducing his equation) or would he have lost interest in it, just as he did for almost all evolutionary subjects to which he applied his talents?

Asking this question allows us to better appreciate Maynard Smith's contribution. It is certainly not for nothing that he was the first author of the ‘Logic of animal conflict’ [1]. He solved computer problems that frustrated Price (see below), extended the analysis to the ‘War of Attrition’ and wrote the paper. ‘The logic of animal conflict’ investigated two models of contests. The main model was a computer simulation involving five strategies played against each other over a number of moves. This model later provided the basis for the simplified, one-move ‘Hawk–Dove’ game. In the second model, the War of Attrition, both opponents continue displaying or fighting until one retreats. The essential difference is that, in Hawk–Dove, costs (e.g. a serious injury) are discrete and sustained by only one opponent when both play Hawk, whereas in War of Attrition costs increase continuously for both opponents during the contest until one gives up. The main aim was to demonstrate that ‘Retaliator’, a strategy of limited aggression, can arise through individual selection; the formulation in the published version reflected a compromise between Maynard Smith's continuous advocacy of individual selection (versus group benefit), and Price's long-held view that ‘possibly many adaptations that appear to be group-benefitting and not individual-benefitting will turn out on deeper analysis to be both individual- and group-benefitting’ (Price, ‘Proposal to the Science Research Council’, GRPP 84116, *op. cit.* above; see [33] in the present issue for a more detailed discussion of the 1973 paper [1]).

More than this, Maynard Smith made use of the method. While Price's intelligence can be compared with a bushfire, moving from one field to another, Maynard Smith, who could be similarly versatile in his interests, decided to put EGT to work. In 1974, he developed a more extensive analysis of animal conflicts [34] and began work on the theory of asymmetrical contests (see §4). Around 1975, with Eric Charnov and Jim Bull, he applied EGT to the evolution of hermaphroditism (see §9). By 1977, inspired by unpublished work started by his colleague Paul Harvey, he used EGT to model problems of parental investment, such as when it can pay to desert one's reproductive partner [35]. A vast variety of problems in trait evolution were thus amenable to the simple phenotypic EGT modelling approach. By 1979, Maynard Smith was able to enumerate eight different problems that had already been treated with game theory [36], including inter-species competition, animal dispersal, intra-familial conflict over parental care, resource allocation in plants, hermaphroditism and the evolution of anisogamy. Three years later, his monograph *Evolution and the theory of games* [37] not only provided a general review of the field, but also contained many new developments. It succeeded Maynard Smith and Price's 1973 paper [1] as the main reference on the subject. From a method limited to studying animal contests, Maynard Smith had made EGT a general framework for studying selection on phenotypes.

Discussing Maynard Smith's theoretical contributions would deserve a separate treatment. For instance, it would be of interest to examine his definitions of ESS and how he revised them in view of later development of stability analysis. Our aim here is rather to discuss his other effect on the growth of this field, as a powerful catalyser and disseminator. The mechanisms of his influence were subtle, and even paradoxical.

In the early 1970s, Maynard Smith was the head of the School of Biological Sciences at the University of Sussex. He and his colleagues Paul Harvey, Brian and Deborah Charlesworth and Timothy Clutton-Brock constituted the nucleus of a very active group in theoretical and empirical biology, with a vibrant social life, vividly described by Marek Kohn [38]. In the 1970s, this group became a hotspot for theoretical biologists. However, Maynard Smith did not found a school of game theory at Sussex, or a centre of research entirely devoted to the study of animal conflicts. He showed limited interest in attracting funds for his work [2], and visitors were expected to find their own financial support. He even avoided accepting students intending to work specifically on game theory. When Michael Rose asked him to act as his advisor while working on theoretical biology, Maynard Smith declined and asked him to work with his colleague Brian Charlesworth [39]. Researchers on sabbatical at Sussex in those years do remember an extremely stimulating environment, but not a frantic hub of game theory.

Why then do we argue that Maynard Smith exerted a pivotal and continuous influence over the growth of EGT? First of all, because of his talks and conferences. One can almost track the diffusion of game theory into evolutionary biology by following his talks; he became, for a time, both the main model-maker and the itinerant popularizer of the methodology. Maynard Smith was a very charismatic orator. ‘I had read his scientific papers before attending the conference’, Michael Rose remembered, ‘but they had not prepared me for his verbal powers. John could captivate an audience of scientists like Elvis Presley singing to a Las Vegas crowd’ [39, p. 5]. It is a testimony to Maynard Smith's unusual ability to capture the attention of his audiences and convince them of the fertility of his area of study that three mathematicians we contacted for this paper acknowledged that their interest in EGT was a direct outcome of attending a Maynard Smith lecture. Tim Bishop, perhaps the first student in mathematics to devote a PhD dissertation to EGT, attended a UK mathematical genetics conference in early 1975 where Maynard Smith described his result on the mixed ESS for War of Attrition contests. His informal proof was enough to convince Bishop's advisor, the Sheffield mathematician Chris Cannings, that a more formal treatment was needed, setting the subject for Bishop's PhD dissertation. Both Bishop and his advisor, went on to work extensively on the war of attrition. Two other prominent examples of mathematical converts are W. G. Hines and Peter Taylor, who also made major contributions to ESS theory in the 1970s and 1980s. They both first heard of EGT when Maynard Smith lectured at a conference of the Canadian Society of Mathematics in 1975 and were sufficiently impressed to turn to EGT (see §7).

Last, but not least, we should mention Maynard Smith's ability to orientate the field through his refereeing works for science journals. Being one of the few biologists able to assess modelling work in the United Kingdom, he was frequently asked to review manuscripts on theoretical biology. With his rising reputation, he became even more central: in game theory, most papers were directed to him, either by journals for reviews or by researchers for comments. He could thus relate results obtained by researchers from different schools and help information to circulate between them. Several theoreticians have told us of their feeling of relief when reading his reviews, making clear the problem and the main points of the papers, sometimes lost in a mass of complex algebra, to the benefit of the author. But reviewing papers also helped Maynard Smith to follow EGT developments, presenting him with new ideas and stimulation to think about new questions, or potential for collaborative ventures with other scientists. It is to these other actors that we now turn our focus. We consider who used the methods developed by Maynard Smith and Price, what were their motivations, and how their work affected the growth of the field.

## 4. Turning to theory, making models more realistic

Let us begin this survey with a personal example. In my PhD at the University of Bristol, I had made a general investigation of sexual selection in dung flies. Males gather around fresh cattle droppings to mate with and then guard gravid females as they lay their eggs in the dung. Over the course of this work, I observed males fighting for females [40,41]. Soon after, during the 2 years before publication of Maynard Smith and Price's 1973 paper [1], I had started work on a theoretical analysis of animal contests, based on individual selection and on the notion that contestants assessed asymmetries between them. This paper was in draft when I read Maynard Smith and Price's paper and its synopsis [26] in 1973.

Depressed by the news (it is never great to learn that one of the best theoretical biologists in the country has just published a paper on the very same problem), I nonetheless noticed significant differences in our emphases. Maynard Smith and Price had assumed symmetry; the two contestants were equal in all respects. In my approach, I had emphasized that contestants were not equal. I had observed numerous fights between male dung flies, where asymmetries between contestants were generally obvious (e.g. ‘owner’–‘attacker’, larger–smaller). In my view, models needed to include this major feature. I distinguished between two main kinds of payoff-related asymmetries between contestants. The first concerns fighting ability, which I called ‘resource-holding power’ (RHP; thinking of male flies keeping hold on a female against competitors): animals differ in relative strength, which should affect contest outcome. The second asymmetry concerns the value of the resource (*V*): a food item, mating opportunity, or a territory cannot be assumed to be of equal value for all contestants. In essence, my view was that fighting functioned to assess these relative RHPs and *V*s, which determined relative fitness payoffs, and thus how long each contestant could ‘afford’ to fight. This analysis resulted in an ‘assessor rule’ for contest outcomes, relating to the benefit/cost ratios of the two opponents. Maynard Smith reviewed the paper, found it interesting, and investigated a different case: a situation where asymmetry between contestants was *not* related to payoffs. He showed that when fighting can lead to dangerous injury, a purely arbitrary (i.e. payoff-uncorrelated) asymmetry between otherwise symmetric contestants could be used to define a ‘peaceful’ (i.e. non-escalatory) solution. He published this result in the same issue of *Journal of Theoretical Biology* in 1974 [34], immediately before my paper on assessment strategies [42].

Maynard Smith invited me to Sussex for discussions in July 1974, and we corresponded on the topic for two years. A more substantial account has been given elsewhere [41] of our subsequent collaboration, which applied ESS logic to contests in which contestants have asymmetric costs and benefits of fighting (i.e. payoff-related asymmetries) [43]. Although at that time I had limited mathematical expertise and was unable to contribute significantly to the mathematical developments, I sent several letters to Maynard Smith during 1974 suggesting various lines of enquiry, a few of which were followed up in our joint paper, published in *Animal Behaviour* in 1976 [43]. Theoretical papers on contests quickly followed by other authors (e.g. [44–48]).

One of the major developments of this area was the sequential assessment game constructed by Magnus Enquist and Olof Leimar [49]. This model develops the notion that RHP assessment is not immediate, but improves during the contest: animals increase their information about their relative RHPs during successive bouts in a contest. Although it could be claimed that some of its essence had been foreshadowed earlier [42,43,50], their very plausible analysis led to detailed and specific predictions amenable to quantitative tests. Indeed, Enquist later decided to test them empirically (with success) by devising contests in aquaria between males of the cichlid fish *Nannacara anomala* [51].

I can draw some similarities between Enquist and my own attitudes to Maynard Smith and Price's 1973 approach. We were both attracted by EGT's potential to develop a valid theory of animal contests in terms of individual selection, and both had to become more mathematically proficient to manipulate ESS methods. While a graduate student at the University of Stockholm in the mid-1970s, Enquist had also discovered Maynard Smith's book *On evolution* [26]. Enthusiastic, he started a PhD on animal contest theory (for which I later became external examiner). There was no mathematical expertise on biological issues among students of animal behaviour at Stockholm at that time, so he had to train himself in mathematical biology. He asked Olof Leimar, then a PhD student in theoretical physics, for assistance; Leimar became so interested in EGT that he too switched his PhD to the subject. Similarly, following Maynard Smith's advice, I taught myself some basic skill in calculus to use ESS methods.

However, Enquist and I also reacted to what we perceived as gross simplifications in the first models, which, in our view, lacked biological realism. I have mentioned my unease about Maynard Smith & Price's assumption of symmetry, which conflicted with my intuition derived from dung fly contests. Similarly, Enquist was extremely critical of the Hawk–Dove game. As an amateur naturalist since boyhood, he felt that the Hawk–Dove game did not capture how animals fight. It was only with time that he came to appreciate its value as a guide for clarifying thinking. What we wish to emphasize here is that researchers can be attracted to a method because of its perceived deficiencies: they then feel they have something to offer.

Consultation of Maynard Smith's and Price's papers held at the British Library shows in retrospect that Enquist and I were in good company in questioning the realism of the early Hawk–Dove models. It transpires that Maynard Smith and Price had themselves been worried by the relevance of game theory to the analysis of conflicts in real animals. Price was anxious to get his facts right and searched for appropriate references in the empirical literature (G. R. Price to V. Geist, 24 March 1974, JMSP). Similarly, Maynard Smith consulted his ethologist friends for advice, but with limited success. On their own admission, his former colleagues at the University of Sussex were unimpressed (P. Slater 2011, personal communication to J.B.G.): there seemed to be a huge gap between the complex behaviours studied by ethologists and the theoretical analyses using simplified strategies. But for others, this gap was a stimulation to embrace theoretical biology.

Models and further observations have challenged the basic statement Price wanted to demonstrate with game theory, that natural selection would usually lead to peaceful settlements of contests. Animals do fight and sometimes at great cost to themselves (e.g. see [52–54]). Furthermore, 50 years later, some theoretical results are still challenging biological intuition. Maynard Smith's 1974 demonstration that contestants could use ‘uncorrelated’ asymmetries to peacefully settle conflicts [34] is the kind of result that leaves me ambivalent, as a theoretical biologist. My natural history intuition tells me that this is unlikely, and I think Dan Rubenstein and I managed to show that it cannot occur in a War of Attrition [50] when individuals can accurately assess payoffs (see also [48,55]). But as a theoretician, I cannot disagree with the formal proof that it may apply in Hawk–Dove situations.

## 5. Evolutionary game theory and the rise of behavioural ecology

While ethology had mostly been preoccupied with describing an animal's behaviour patterns, behavioural repertoires and the internal system of ‘drives’, or internal states evoking them [56], the new science of behavioural ecology focused on adaptive value and represented a major change in approach (see Stuhrmann's detailed account [57], and also [58,59]). Group selection interpretations of adaptation, often implicit, were pervasive in ethology and ecology up to the late 1960s and beyond, until George Williams' famous critique favouring individual selection in 1966 [60], after which debate continued (e.g. [61,62]). From very early on, students of behavioural ecology saw EGT as relevant to their data, and, more broadly, as giving direction to their fieldwork (e.g. see [63]). More than that, it exemplified the research programme of studying behaviours as adaptations fashioned by natural selection. The theory was rapidly popularized. Although E. O. Wilson's *Sociobiogy: the new synthesis* [64] in 1975 only included Maynard Smith and Price's 1973 result as a hypothesis on ritualized aggression, behavioural ecologists based in the UK laid stress on optimality and ESS approaches. No specific chapter was devoted to it in the first edition in 1978 of Krebs and Davies' *Behavioural ecology: an evolutionary approach* [65], but the cover shows a hawk chasing doves and the Hawk–Dove payoff matrix, and the editors stressed that the method underlay (or should underlie) arguments in several chapters, including sex ratio, lek behaviours, ritualized conflicts and animal distributions. Richard Dawkins in *The selfish gene* [66] in 1976 promoted EGT as one of the major developments in twentieth century science. In accord with the enthusiasm of the times, I (in the second edition of *Behavioural ecology* [67]) praised it as the major recent development in evolutionary theory. In a nutshell, EGT found its place among the three major theories available in behaviour studies, alongside inclusive fitness and (frequency-independent) optimization theory.

Two major places for the spread of new theories among students of animal behaviour were certainly the universities of Oxford and Cambridge in the United Kingdom [57]. Documenting the flux of information from and to these places certainly sheds much insight into the rapid growth of behavioural ecology [57]. With a strong basis of trained ethologists and population ecologists, both held vibrant seminar series to which leading researchers, such as Trivers and Maynard Smith, came to present their recent works. Students there were informed of upcoming work and were able to develop research strategies accordingly. Thus at Oxford, Nick Davies designed ingenious experiments on the speckled wood butterfly, testing the ‘owner win’ rule of animal contests [68]. While being the main fortress of the more ethological approach, keeping proximate factors under close focus, Cambridge also became an important centre of exchange. From 1975 to 1980, Patrick Bateson assembled a Sociobiology Research Group at King's College, which allowed considerable exchanges and collaborative ventures between students of animal behaviour [69]. For example, Tim Clutton-Brock (later at the University of Sussex) and his co-workers were studying red deer fights, and discussing them in terms of EGT predictions [70,71].

The King's Sociobiology Group was certainly important for me. Based at Liverpool, I was (relatively) isolated from the main lines of ongoing research in the nascent behavioural ecology, and depended upon correspondence, reviews and visits to follow progress. A one-year stay at Cambridge (1978–1979) was an opportunity to start new collaborations. I collaborated with Dan Rubenstein on modelling assessment of asymmetries in contests [50], and analysed data on struggles between male dung flies for females with the statistician Elizabeth A. Thompson. Although the distribution of dung fly contest lengths seemed roughly consistent with a symmetric war of attrition, it became clear that they were more likely to be asymmetric conflicts in which the outcome favoured the ‘owner’ [72], as Hrefna Sigurjónsdóttir (then my PhD student) and I later demonstrated [73].

Let us consider in more detail the examples of two US researchers, Jane Brockmann and Susan Riechert, whose career paths shed light on circulation of information to researchers in North America. Both trained at the University of Wisconsin in the late 1960s to early 1970s, where Brockmann studied the behaviour of golden digger wasps, *Sphex ichneumoneus*, while Riechert specialized on the ecology of the spider *Agelenopsis aperta*. Both of their works had initially a strong ethological bent; they used quantitative ethological methods such as analysis of behaviour sequences. Only in later phases of their work did they reinterpret their data in the light of EGT, providing among the best empirical applications of this approach (see §6).

Personal interactions mattered in the circulation of the approach; Brockmann's and Riechert's foray into EGT depended crucially upon such interactions. After her PhD, Brockmann undertook a sabbatical in Oxford in 1977–1978. She had planned to work with David MacFarland, then the leading expert on quantitative methods in ethology; MacFarland being absent, she instead collaborated with Richard Dawkins, who had promoted EGT in *The selfish gene*. They jointly used ESS methods to reanalyse her observations on digger wasps (see §6). Later, Brockmann put Riechert, who was making a transition from ecology to behaviour, in contact with John Maynard Smith to reanalyse her own data on spider contests. Through Brockmann's intercession, Riechert launched an influential collaboration with Maynard Smith's former student, Peter Hammerstein, which led to a major attempt at measuring payoffs in natural populations, over a field study spanning decades (see enlightening accounts of this important work in [74–76]).

## 6. A method for behavioural ecology: a case study

Both Brockmann's and Riechert's pathways testify to the importance of collaborative ventures in this first flurry of EGT application by fieldworkers. To analyse one's data in EGT terms, collaborating with colleagues better versed in mathematical analysis was often critical.

Brockmann's collaboration in Oxford with Richard Dawkins and the then fledgling theoretical biologist Alan Grafen is particularly instructive of the lessons gained with these new tools.

Studying the nesting behaviour of golden digger wasps in North America during her PhD, Brockmann investigated joint provisioning between wasps. Female wasps nest in underground burrows, which they usually dig and provision solitarily, but occasionally two females occupy the same burrow and fight whenever they meet; ultimately only one lays an egg in the shared nest, which benefits from the work of both provisioners. Brockmann interpreted this as a possible example of the evolution of social behaviour: females cooperate in establishing resources that are later monopolized by the winner. This situation offered a direct example of a primitively social behaviour leading to the evolution of eusociality, then the main ‘obsession’ of Hymenopteran sociobiology [77].

At the time of her PhD, Brockmann lacked a clear theoretical or modelling framework for analysing her data in such a way. She tried to determine the payoffs, but lacked a way forward. Reading Dawkins' *Selfish gene* [66] on the plane to England in 1977 for her Oxford sabbatical, she realized it contained several ideas that might apply to the unpublished chapter on joint provisioning in her dissertation. She learnt more about EGT in conversations with Dawkins during her Oxford sabbatical, and they collaborated on a joint analysis of her data, which we detail below, enlisting the recently graduated Alan Grafen, who had decided to pursue a further degree in economics instead of biology. Dawkins wanted to give Grafen a project that would keep him in his field. The *Sphex* study provided the lure.

Their process of model building and data interpretation was collaborative: Brockmann provided the empirical insight and data for calculating payoffs, Grafen formulated the models and performed calculations, and Dawkins and Brockmann wrote the manuscript [78]. As an editor of *Animal Behaviour*, Dawkins had developed his own views on how to write a scientific paper. Rather than giving a rag-bag of ‘Methods’ encompassing several experiments followed by a similarly unstructured list of ‘Results’, he encouraged authors to write their hypothesis first, their experimental protocols, their conclusion, before turning to the next hypothesis and experiment [79]. The *Sphex* collaboration gave him an opportunity to apply his editorial recommendations. The paper, processed in record time by Maynard Smith for the *Journal of Theoretical Biology,* explains very clearly how they initially applied Brockmann's interpretation and formulated it in EGT terms, before rejecting it in favour of an alternative model with a different assumption.

Their line of attack was as follows [78]. Assuming that burrows are more successful when two females collaborate, they distinguished between two different strategies for a wasp: (i) digging and founding a burrow alone, and (ii) actively joining a founder's burrow and contributing to provisioning it, in the prospect of gaining control of the burrow later. To exist as a mixed ESS, both strategies should achieve equal payoffs in a frequency-dependent equilibrium. After analysis, they had to discount this model: founders did approximately twice as well as joiners. There was obviously little benefit in joining a nest once another female had begun provisioning it. Brockmann's original intuition of making it a model of social behaviour had failed (for her views on joint nesting as a preadaptation to social life, see [80]).

To account for her data, they reconsidered the role of proximate factors, especially the wasp's ability to assess the volume of larval food material in a burrow. They had assumed that a wasp knew as much as the scientist observing it: when entering a burrow, it could assess whether the burrow was already being provisioned by another female. Dropping this assumption, they considered the possibility that wasps provision burrows without ‘knowing’ whether they are provisioned or not. Then, as they note, ‘sharing’ a burrow is a regrettable consequence of having entered and provisioning a burrow. Their revised model worked for one of two populations (New Hampshire), but not the other (Michigan) [78].

Brockmann *et al*.'s study [78] is one of many that demonstrate how optimality theory (which includes EGT) can be employed to test hypotheses about adaptation. It is not a procedure for demonstrating that a trait is optimal, which is an assumption of the method; rather, should observations match model predictions, it suggests that the researcher may have correctly identified the selective forces operative in shaping the trait [81]. In Brockmann *et al*.'s case, EGT effectively changed their interpretation of field data. Accurately formulated model assumptions and predictions could be compared with empirical evidence, allowing field researchers to accept (or reject) their hypotheses. This study was praised by researchers anxious to raise standards of empirical tests of optimality theory in evolutionary biology [82].

However, the paper also showed why EGT and optimality theory were not magic keys applying to any population. A model applying well in one population did not necessarily work in another. For their non-fitting population (Michigan), Brockmann *et al*. limited themselves to conjecturing the presence of gene flow from other populations. Interestingly, like Brockmann *et al*., Hammerstein & Riechert [74] had mixed success in their long-term comparative study of the spider *A. aperta* living in different environments. They found a close fit with EGT predictions for one ecotype (a desert grassland population), but not for a second (living in a more favourable riparian habitat). They suggested that gene flow prevented this second population from completely adapting to its local environment. This explanation is plausible: Riechert and Maynard Smith showed that the two ecotypes differ genetically [83], and that there is indeed evidence of high gene flow in the second population [84]. This extensive line of work is a helpful reminder that, while apparently circumventing information on the underlying genetics, the empirical success of predictions based on EGT critically depends upon the opportunities, and constraints, of genetics (see §§8 and 10).

## 7. Beyond behavioural ecology: interdisciplinary collaborations

Maynard Smith and Price's [1] paper had developed EGT as a technique for modelling animal contests. For some years, contests were indeed one of the main areas of its application. However, researchers quickly realized that EGT had much broader applications. Research went in two directions. The first—applying it to other biological problems—was remarkably fruitful. Although several studies had foreshadowed EGT, having a simple formalism energized its rapid application to a burgeoning variety of adaptations (see §3). The second direction involved exploring the mathematical underpinnings of the method. This aim attracted a significant number of applied mathematicians. Theoretical developments in this field were thus published not only in such journals as *Animal Behaviour* or *Journal of Theoretical Biology*, but also in outlets such as *Advances in Applied Probability*.

What did EGT have to offer to mathematicians? Although it seemed simple, ESS theory was rich in hidden complexities. Its simplicity misled the first researchers who used it. A well-known example is Maynard Smith and Price's's analysis of their own game, which was questioned by geneticists from the University of Birmingham, who showed that a new ESS could be found if the Maynard Smith–Price matrix was restricted to a sub-set of strategies [85]. Happy with the main results, which were consistent with their general interpretation of animal contests, Maynard Smith and Price did not perceive that their own simulations were richer than initially planned.

More fundamental problems appeared when Maynard Smith and his collaborators tried to delineate the method more clearly. One of the two mathematicians drawn to EGT by Maynard Smith's talk in Canada (see §2), W. G. Hines, was initially deceived by ESS's apparent simplicity. As a statistician, he initially believed that EGT was sufficiently well established for him to build methods for estimating payoff matrices from field data. He soon realized that clarifying its mathematical foundations was still a work in progress. As he later commented: ‘it seemed to me that a field of study motivated by a wish to understand issues in theoretical biology became replaced by interest in an enjoyable diversity of questions that arose from that wish, but which gained academic lives of their own’ (Gordon Hines 2011, personal communication to J.B.G.). It was not clear, for example, whether a system must have an ESS, or how many ESSs could coexist for a given system. Nor was it clear how to find all the ESSs. And the very meaning of an ESS was obscure. For instance, was a mixed ESS a property of the population, with individuals playing possibly very different strategies, or a given set of strategies played by each individual in a population? The first investigations on these issues began in Sussex. John Haigh, Maynard Smith's applied probability colleague at the University of Sussex, studied the mathematical properties of ESS in *m*×*m* matrices (*m* being larger than 2), showing that it was possible that no ESS existed, and deriving methods for finding all ESSs in a matrix [86]. Also, would a population actually converge to an ESS (e.g. see [87])? In the decade that followed, the mathematical study of evolutionary stability became a field of inquiry of its own (see summary by [88]).

A major contribution was made by Peter Taylor, the other mathematician converted by Maynard Smith during this talk in Canada. Interested in foundational issues, Taylor was struck by an apparent anarchy: Maynard Smith and his collaborators had built different models for each situation, but was there any real mathematical unity behind them (Peter Taylor 2011, personal communication to J.B.G.)? He started to compare different ESS methods, such as Maynard Smith's and similar methods in sex ratio evolution (see §9), striving to make it a coherent body. Taylor's solution was to incorporate EGT into the mathematical theory of dynamical systems. Here, a strategy's payoff is essentially proportional to the growth rate of those who adopt it in a population. This work uncovered major similarities with other areas of biological research. Following Taylor & Jonker's equation for game dynamics [89], Schuster & Sigmund [90] showed how the same basic replicator equation applied to four different research areas (population genetics, ecology, animal behaviour and prebiotic evolution), thus integrating EGT into the realm of ‘replicator dynamics’.

What we wish to emphasize is that, just as EGT went beyond the theory of animal contests, it emerged as much more than a theory for behavioural ecology. It was a genuinely interdisciplinary field, to which mathematically orientated researchers from varied disciplines contributed.

The exchanges with economists were more subtle, as expertly discussed by Grüne-Yanoff [91]. There were unquestionably interactions between economics and behavioural ecologists in the 1970s and early 1980s. The first major dialogue, and first EGT conference, was organized in Bielefeld in November 1978 by Peter Hammerstein, a young mathematician working as a theoretical biologist at the Institute of Mathematical Economics (see [76]). In addition to Maynard Smith and the game theorist Reinhard Selten (Nobel Memorial Prize for Economics 1994), delegates included several behavioural ecologists, such as Nick Davies, Richard Dawkins, Alan Grafen, John Krebs and myself. Certainly a major event in EGT, it was followed by conferences in Queen's University, Ontario in 1982 and again in Bielefeld in 1985. Discerning effects from conferences is a difficult undertaking. The 1978 Bielefeld conference was especially important for me: my first international conference, it enabled me to discuss my (then in press) arms race model [92] with Dawkins and Krebs, who were also working on arms races in evolution [93]. My model yielded no ESS, and Selten outlined how his ‘trembling hand’ theorem could be used to stabilize it, which I later used [94]. Though I do not remember the conference as a major basis for collaborations, a notable effect was certainly visible on the organizer, Peter Hammerstein, who cleverly enlisted Selten and Maynard Smith as advisors for his PhD.

However, the short-term effects of these interdisciplinary encounters should not be over-emphasized. To my recollection (and Grüne-Yanoff makes the same general point [91]), we (the biologists) found in EGT a convenient way of framing issues, but were mostly unaware of game theory in economics. Suffice it to quote Maynard Smith's response as to why he never cited Nash: ‘Who is Nash?’ (see [11]). For their part, economists using game theory sometimes perceived EGT as redundant: that it could offer a genuinely different and rewarding approach was not immediately apparent, though links grew later and continue (e.g. [95]). Economists discussing EGT spent much effort (sometimes justifiably) relating biologists' discoveries to previous treatments by economists. EGT's main effects on economics were probably felt well after the early 1980s, possibly in the wake of interest generated by Axelrod and Hamilton's celebrated computer simulations of the Tit-for-Tat effect, which turned economists' attention to the issue of equilibrium selection [96]. Before this, it is difficult to pinpoint major collaborations or effects. This was precisely why Maynard Smith was impressed by Selten; according to Hammerstein, he was the first economist who did not try to demonstrate to him that EGT was just another way of doing classic game theory, but understood that it had different aims [76].

An effect of the field's uptake by mathematical modellers trained in mathematics, economics and physics meant a sharp rise in mathematical standards. Maynard Smith often remained, for these mathematicians, the biologist to be consulted over the plausibility of a model's assumptions. However, he sometimes struggled with the increasingly technical developments of this literature (Maynard Smith to Eshel, 3 December 1980, JMSP, Add. MS 86597 A). To the mathematician Christopher Zeeman, he admitted (Maynard Smith to E. C. Zeeman, *ca* 1978, JMSP, Add. MS 86749):

I'm afraid I missed a lot of features of the Hawk-Dove-Brute game. I had no idea its behaviour was so rich when Price and I invented it, but am gradually understanding it. … I find it hard to judge the mathematical interest of all this – it amuses me. Biologically, I suspect the important thing now is for people to look at animals and see whether they have read my papers.

## 8. Evolutionary game theory and population genetics: controversy

Maynard Smith's success in raising EGT's profile represents an interesting puzzle. As a theoretical population geneticist, his influence was sufficiently far-ranging in his field to attract interest among his colleagues. However, population geneticists' contributions to EGT were perhaps more limited than expected: why did so few contribute to EGT in the late 1970s and early 1980s?

A possible reason may well be their lack of interest in it. Population geneticists were then busy with the many problems raised by molecular data, available through the spread of electrophoretic methods. Detecting unambiguous signals of natural selection at the molecular level was very difficult (e.g. see [97]). Phenotypic selection seemed of less pressing concern. An example is Motoo Kimura, arguably the leading theoretical population geneticist at the time. Kimura reacted enthusiastically to Maynard Smith and Price's [1] paper, which he read with ‘absorbing interest’, and sent congratulations for an ‘outstanding achievement’ (Kimura to Maynard Smith, 20 November 1973, JMSP 86726). Kimura was not particularly prone to over-emphasis about matters unrelated to neutrality—his words can be taken at face value. Had this paper been published 10 years earlier, Kimura might have wanted to work more on the subject, as he was always on the lookout for interesting biological problems. But after 1968, he was focused on developing and defending his ‘neutral mutation–random drift’ hypothesis on molecular evolution; it left little room for explorations in other fields.

Further, EGT seemed in part redundant in view of well-researched areas in population genetics. For example, the eminent Nottingham population geneticist Bryan Clarke expressed the view to me that we already had frequency-dependent selection (a concept he had had a major part in developing [98]), so why did we need ESS? Another objection was that EGT gave only a simplified understanding. Using Alan Grafen's provocative turn of phrase, EGT/optimality procedures are based on a ‘phenotypic gambit’ [99]: they study adaptations ‘as if there were a haploid locus at which each distinct strategy was represented by a distinct allele, as if [relative payoffs] gave the number of offspring for each allele, and as if enough mutation occurred to allow each strategy the opportunity to invade’. These simplifying assumptions expel complexities arising through diploid genetic machinery [79, pp. 63–64]. Understandably, it became controversial among professional experts dealing with these complications.

R. C. Lewontin provided a compelling example of this complex reception. As mentioned (§2), in 1961 [12] he had used game theory to tackle one of the major population genetics problems of the day—polymorphism as an adaptation to changing environments. By the mid-1960s, he had lost faith in it, as part of his disillusionment with optimality methods used extensively in ecology for their lack of dynamical sufficiency: optimality only indicates the local optimum for a population, not whether a given population reaches that equilibrium. Lewontin also noted that fits between model predictions and given observed traits can be mere coincidence [100].

In the early 1970s, Maynard Smith informed Lewontin of the new potential of game theory, now refashioned as ESS theory. Lewontin knew Maynard Smith from symposia on theoretical biology organized by C. D. Waddington at the Villa Serbelloni, Lake Como in 1966–1967. High power intellects with very broad interests, superb speakers and debaters, well versed in Marxist views on science and society, they became friends. Lewontin invited Maynard Smith to the University of Chicago, and in 1972 Lewontin spent a term at the University of Sussex, where he wrote chapters of his book *The genetic basis of evolutionary change* [101]. Perhaps less known is that he worked with Maynard Smith on basics of ESS theory. In retrospect, this is not as surprising as it may seem. Closer in spirit to the framework of population genetics than simple optimization methods, ESS concerns analysis of stability against rare mutant strategies arising in a population. According to reports Lewontin wrote to his sponsor, the Atomic Energy Commission, he was able to prove during his stay that a mixed strategy ESS always exists when two pure strategies occur at equilibrium (Lewontin,‘ A study of mathematical models of mutation and selection in multi-locus systems', AEC contract, no. AT(11-1)-1437). Since this work remains unpublished, we do not know exactly what Lewontin demonstrated and must content ourselves with this statement that he worked on such problems. The same reports mention further investigations on game theory. In 1975–1976, a visitor from Israel in Lewontin's laboratory at Harvard, Ilan Eshel, worked on ESS stability criteria (on Eshel, see §10). Eshel was attempting to derive an exact genetic basis for the principle of equalization of parental investment between male and female offspring. Of particular interest to Lewontin was that Eshel's results from ESS analysis ‘rarely’ corresponded to stable equilibria in genetic systems (Lewontin, AEC contract, no. E(11-1)-2472). From then on, Lewontin must have concluded that EGT models gave unreliable conclusions and made his concerns explicit in his review [102] of Maynard Smith's book *Evolution and the theory of games*, and in his review [103] of Maynard Smith's textbook *Evolutionary genetics*.

These criticisms reflected a professional inclination by geneticists. Once one is used to modelling genes, it is not easy to return to the level of phenotypes. Studying genes understandably gives evolutionists the feeling that they are the right level for investigating evolutionary problems, and population genetic formalism helps in comparing the effects of various forces (natural selection, mutation, migration and drift) on a given study system. EGT seemed restricted to the study of selection. Since phenomena *other* than selection could change gene frequencies in populations and even drive them to fixation, ESS formalism could seem misleadingly restrictive (see below). For dealing with phenotypes, population geneticists thus preferred honing their own methods, either through the distinguished empirical approaches of ecological genetics, or through the updated methods of quantitative genetics, which was being rejuvenated for detecting selection on continuous characters [104].

Maynard Smith was not shaken by these objections, which he rather considered a research question, and worked on whether EGT and population genetics gave convergent results [105]. He followed the works of Eshel, who, collaborating with Marcus Feldman, investigated conditions under which the two methods give comparable results (e.g. [106]). On the other hand, he refused to make genetics the sole acceptable formal framework in evolutionary theory. To Bengt Bengtsson, a theoretical population geneticist with reservations about the methodology, he admitted frankly (Maynard Smith to Bengtsson, 27 October 1985, JMSP, Add. MS 86604):

I am very hostile to the … view of the world, which says that we cannot discuss the evolution of a trait unless we know the full details of its genetic determination. If true, this would make nonsense, for example, of functional morphology (and, indeed, of most of physiology!). We are never going to know the detailed genetics of animal behaviour. All that ESS theory is doing is to apply the fitness-set approach to phenotypes, specifically when fitnesses are frequency-dependent, and to assume some additive heritability. I agree it is a bit hand-waving, but it is the best we can do.

Behavioural ecologists like myself responded to the attack on EGT and optimality by suggesting that that the different interests of the two disciplines led both to make unrealistic simplifications, but in opposite directions [67]. ESS theorists sacrificed genetic rigour to consider more complex strategy sets. But population genetics modellers themselves constrained the expansion of strategic possibilities in the interest of analytical tractability: their models were of limited use to those of us working on complex sets of behaviours.

## 9. Evolutionary game theory and population genetics: modelling sex ratio

In contrast with the controversy mentioned in the previous section, some theoretical biologists found it convenient to use both approaches in their mathematical endeavours. The study of sex ratio and sex allocation phenomena offers a remarkable example of such joint pursuits. A history of this field would require an alternative paper, dealing with ESS history before the ESS label. This would begin with the evolution of the 1 : 1 sex ratio, originating with Darwin [107], Carl Düsing [108] and several others (e.g. [109]; see [110] in the present issue for a detailed history). Sex ratio evolution was revisited, notably by Richard Shaw in the mid-1950s, author of the celebrated Shaw–Mohler equation (see his autobiography [111]), and for later extensions to cases when the assumptions underlying the 1 : 1 ratio do not hold, initially by W. D. Hamilton [14], see [110,112–114]. We here restrict our attention to Eric Charnov, who adopted ESS methods in the 1970s in his study of sex allocation theory, drawing on recollections he shared with us.

Charnov trained as an ecologist at the University of Washington, Seattle in the late 1960s and early 1970s. As a graduate student, Charnov fell under the spell of V. C. Wynne-Edwards' interpretation of trait evolution through group selection [115]. In Charnov's recollections, Wynne-Edwards asked broad, far-ranging questions going to the heart of biology, and group selection's explanatory power seemed impressive. Then, in 1971, Charnov attended a course given by a former student of David Lack, Gordon Orians, who framed his lectures against Wynne-Edwards' explanations. After digesting these criticisms and reorganizing his thinking accordingly, Charnov concluded that the questions remained, even if Wynne-Edwards’ answers were wrong. ‘Many things, like alarm calls, became puzzles to be thought about. Unsolved puzzles, partially solved puzzles' (E. Charnov 2012, personal communication to J.B.G.). Eager to address these on a firm ground, Charnov turned himself into a theoretical ecologist. After taking several graduate-level classes in economics and optimization methods (operations research), he set out to apply fitness optimization ideas to the study of animal behaviour and life-history evolution. Stimulated by the optimal diet models of R. H. MacArthur, Eric Pianka and John Emlen, he first set to work on theory of optimal foraging, striving to make models amenable to quantitative tests [116].

In summer 1974, then a professor at the University of Utah, Charnov turned his attention from optimal foraging to sex ratio, because the former proved more difficult for estimating tradeoffs and testing predictions. ‘Sex allocation was a [life history] theory that made sometimes surprising predictions and could be tested because we could know the tradeoffs, at least well enough. I was focused on realistic, testable theory from the beginning; and getting data’ (E. Charnov 2022, personal communication to G.A.P.). This shift in research direction was catalysed by a book manuscript on the subject by an empiricist colleague. That manuscript, still unpublished, explained the Shaw–Mohler equation for sex ratio [21]. Building on Fisher's verbal discussion, Richard Shaw and his colleague Dawson Mohler had shown how to model sex ratio by tracking an autosomal gene affecting sex ratio through the offspring and grand-offspring generations. This led to the surprising result, that at the 1 : 1 equilibrium all variants were of equal fitness. In other words, an equal sex ratio is an evolutionarily stable (population) strategy. Although many genetic variants coding for biased sex ratios can coexist in the population, the population equilibrium is 1 : 1. The Shaw-Mohler result is a remarkable example of the patterns that can emerge at the phenotypic level. In the second half of the 1970s, Charnov made it the basis for extensive investigations, asking how it would apply to cases, such as simultaneous and sequential hermaphroditism, not considered by Shaw and Mohler.

It is interesting to note that Charnov used two different methods. On the one hand, he used phenotypic methods. For instance, he rederived Shaw and Mohler's result in the more complex demographic setting of an age-structured population with overlapping generations (the Shaw–Mohler model was designed for separate generations) [117]. After a stay in the UK in summer 1975, Charnov, his PhD student Jim Bull, and Maynard Smith published a joint paper, which presented a new quantitative theory for hermaphroditism [118]. Based on their independent derivations, the paper was written by Maynard Smith and featured an ESS model. ‘His argument was cumbersome, but correct’, Charnov granted (E. Charnov 2022, personal communication to G.A.P.). He then turned to theory of sequential hermaphroditism and applied it to empirical data. The best data available were provided by marine ecologists. Charnov thus used extensive data on life-history parameters in a pandalid shrimp (a protandrous hermaphrodite) to investigate timing of sex change [119].

But his stay in the UK had helped Charnov to extend his range of modelling tools. At the University of Sussex, he interacted with Brian Charlesworth, an expert at anchoring theoretical developments into firm population genetics foundations. Charlesworth taught Charnov how to analyse stability in population genetics equations, by investigating the effect of introducing into a population a mutant at a single locus. Back in Utah, Charnov decided to expand his modelling toolkit and retrained in population genetics, analysing scores of examples, including in sex allocation theory. There are good reasons for not neglecting genetics. Among many animals, sex determination depends on genetic mechanisms, such as homogamety–heterogamety. Running computer simulations with Bull, Charnov investigated how mutants modifying sex determination affected the population polymorphism, and, in turn, led to new sex-determining systems. It was in this context that they appreciated the power of Shaw and Mohler's result. In his own words, ‘when Bull and I did our simulations, we were very confused. Every time we set a starting frequency the genotype frequencies changed for, maybe, 6 generations and then just stopped. And for the same genotype system, where it stopped depended completely on the starting frequency. It took us a while to realize that the equilibrium genotype frequencies depended entirely upon starting frequencies, BUT EVERY PHENOTYPIC EQUILIBRIUM WAS A POPULATION SEX RATIO OF 1/2. I had never encountered a dynamical model like that’ (E. Charnov 2022, personal communication to G.A.P.). (Unaware of all previous sex ratio theory, I had encountered exactly the same problem in 1967 during my PhD when running computer simulations on sex ratio evolution; see [17]).

By the late 1970s, Charnov decided to summarize this effort by investigating various patterns of genetic sex determination, showing in each case how to recover the Shaw–Mohler equation as a guiding principle [120]. In his view, although population genetics methods (invasion dynamics) were seen as more rigorous, and although they were necessary in investigation of complex patterns when the fitness function cannot readily be specified, such as haplodiploid inheritance, the Shaw–Mohler method was often much easier for obtaining the phenotypic answers he sought, and it helped focus on the quantities of interest. In conversation with us, Charnov recollected that Lewontin served as referee for his tenure application. While Lewontin praised Charnov's work for putting sex allocation theory on a secure population genetic basis, Charnov tended to view this work as being derivative rather than foundational. The genetic methods had mainly confirmed the phenotypic answers.

## 10. Ilan Eshel, the timescale of the evolutionary process and conclusive comments

Although there was controversy, these discussions between theoretical biologists and population geneticists should not be framed too exclusively as confrontation. Showing convergences (or differences) between EGT and population genetics models was a widely shared preoccupation within the small community of EGT theoreticians in the early 1980s. For instance, Hines determined that population means tended to the ESS (when possible) for the single-locus multi-allele case with additive inheritance [121], before investigating ESS under more complex genotypic maps with Bishop (e.g. [122]). This should be no surprise. Comparison between rival modelling methods is commonplace in the history of science, and forms a significant part of assimilating ideas and gaining confidence in new methods. A prominent example is given by the history of calculus in the eighteenth century, split between continental methods inspired by Leibniz's calculus, and geometric methods in England in continuity with Newton's approach in the *Principia* (see [123]).

A different line of attack was Ilan Eshel's sustained attempt at framing population genetics and phenotypic approaches as alternative (but compatible) perspectives focused on distinct processes. Trained as a mathematician in Israel, Eshel obtained a PhD at Stanford for work on the advantages of recombination in a constant environment. He became an important member of the school advocating mathematically ‘exact’ approaches in population genetics theory [124]. However, he also considered the issues tackled by Hamilton, Maynard Smith and the broader field of phenotypic evolution to be the major problems in evolutionary theory. One of his long-standing interests concerned the behaviour of herds facing predators. Inspired by Hamilton's ‘selfish herd’ theory [125], Eshel used EGT to investigate how shared common interests between predators and the strongest or fastest individuals in a group of prey species may result in those prey helping the predators to locate the weakest or slowest prey in the herd [126]. As a theoretician, Eshel thus faced a major contradiction. Phenotypic problems were the issues of evolutionary importance, but conclusions drawn from purely phenotypic approaches, or from simple one-locus two-allele models, were unlikely to hold under more complex genetic situations; conversely, exact population genetic models (being usually limited to two-locus theory) were unlikely to apply to the many important adaptations whose genetic basis was unknown. Building on two decades of work on these issues, Eshel's solution to this conundrum was to draw a distinction between two processes of evolution, which he called short-term and long-term views of evolution (e.g. [127–129]).

Eshel's scheme is reminiscent of Sewall Wright's shifting balance theory of adaptive evolution. Both rely on a process of ‘trial and error’ circumventing limitations of the process of gene frequency change. Wright famously declared that, to evolve, a population should not be under the sole control of natural selection; in his evocative shifting landscapes of gene frequencies, selection leads a population to a single peak, possibly suboptimal, and exhausts variation necessary for further evolution. According to Wright, another process, based on differentiation of the species into partially isolated subpopulations, is required for exploring the full adaptive landscape, for reaching the highest peaks and for retaining variation [130]. In Eshel's scheme, the ability of populations to reach a phenotypic optimum (or, in frequency-dependent selection, an ESS) is bounded by the complications of the genetic machinery in systems with diploid inheritance, especially epistasis and recombination (see [131] for a general history of the problem). However, Eshel argued that gene frequency change under a fixed set of genotypes does not offer a full description of the process of adaptive evolution. It represents only one scale of evolution, which he called short-term evolution.

A longer-term view includes the continuous supply of new mutations. When advantageous mutations are rare relative to the time required to reach equilibrium, new mutations occur away from the stable equilibria of the short-term process (when they exist) and reset the process of gene frequency change towards new states (to a new stable equilibrium, a new cycle or a state of chaos). Long-term evolution proceeds by an infinite sequence of similar transitions, from one fixed set of genotypes to another fixed set of genotypes, each of them being subject to the episodes of short-term evolution caused by the establishment of successful mutations. Eshel's work and, similarly, that of Hammerstein [132] focused on demonstrating that, in the long-term process, a multi-locus genetic system under frequency-dependent selection can approach an ESS (when it exists).

To many, this effectively solved the conundrum. Both approaches were correct for their respective purposes. These were just different purposes, representing different views on the evolutionary process and how to study it mathematically. However, empirically, as Eshel admitted, long-term evolution does not guarantee that any population has reached an ESS. For instance, the supply of newly arising mutations may have been deficient, either because of insufficient time or accidental allele loss (for asymmetric contests, see [133]). Thus, his scheme might mostly have reassured only those of us who were already comfortable with ESS methods, happy to focus on the phenotypes and ready to leave organisms deal with their own genetic problems.

How theoreticians consider unification attempts such as Eshel's scheme is an open question. Grafen, who has developed his own major long-term programme to formalize NeoDarwinism [134], once observed that few biologists seem concerned by foundational issues. ‘It may … be the case that fashion has somewhat turned against ‘high theory’, and favours more low-tech, more empirical work that lacks the taint of master narrative’ [134, p. 63; 135]. As far as I can offer a tentative conclusion based on my own involvement, the years in which I was most involved in the study of animal conflicts—the early days of EGT—were years of ‘low theory’. Certainly, then, EGT meant extending the full range of Darwinism to animal behaviour and many other areas, and this was of major theoretical significance to us. But the mathematics we learnt was pragmatic. Maynard Smith led us to formulate problems and seek solutions, to make models, to learn the basics of calculus, but also—and fundamentally—to gain confidence in ourselves and to trust our instincts.

## Data Availability

This article has no additional data.

## Endnote

^1^Abbreviations used in in-text manuscript references.

JMSP: John Maynard Smith papers, Add. MS 86569–86840, British Library, London.

GRPP: George R. Price papers, Add. MS 84115–84116, British Library, London.

WDHP: William D. Hamilton papers, British Library, London.
